# Functional Analysis of NopM, a Novel E3 Ubiquitin Ligase (NEL) Domain Effector of *Rhizobium* sp. Strain NGR234

**DOI:** 10.1371/journal.ppat.1002707

**Published:** 2012-05-17

**Authors:** Da-Wei Xin, Sha Liao, Zhi-Ping Xie, Dagmar R. Hann, Lea Steinle, Thomas Boller, Christian Staehelin

**Affiliations:** 1 State Key Laboratory of Biocontrol, School of Life Sciences, Sun Yat-sen University, Guangzhou, China; 2 Botanisches Institut der Universität Basel, Zurich Basel Plant Science Center, Basel, Switzerland; Oregon State, United States of America

## Abstract

Type 3 effector proteins secreted via the bacterial type 3 secretion system (T3SS) are not only virulence factors of pathogenic bacteria, but also influence symbiotic interactions between nitrogen-fixing nodule bacteria (rhizobia) and leguminous host plants. In this study, we characterized NopM (nodulation outer protein M) of *Rhizobium* sp. strain NGR234, which shows sequence similarities with novel E3 ubiquitin ligase (NEL) domain effectors from the human pathogens *Shigella flexneri* and *Salomonella enterica*. NopM expressed in *Escherichia coli*, but not the non-functional mutant protein NopM-C338A, showed E3 ubiquitin ligase activity *in vitro*. *In vivo*, NopM, but not inactive NopM-C338A, promoted nodulation of the host plant *Lablab purpureus* by NGR234. When NopM was expressed in yeast, it inhibited mating pheromone signaling, a mitogen-activated protein (MAP) kinase pathway. When expressed in the plant *Nicotiana benthamiana*, NopM inhibited one part of the plant's defense response, as shown by a reduced production of reactive oxygen species (ROS) in response to the flagellin peptide flg22, whereas it stimulated another part, namely the induction of defense genes. In summary, our data indicate the potential for NopM as a functional NEL domain E3 ubiquitin ligase. Our findings that NopM dampened the flg22-induced ROS burst in *N. benthamiana* but promoted defense gene induction are consistent with the concept that pattern-triggered immunity is split in two separate signaling branches, one leading to ROS production and the other to defense gene induction.

## Author Summary

Many Gram-negative bacterial pathogens possess type 3 secretion systems, which deliver effector proteins into eukaryotic host cells through needle-like structures. Effectors manipulate the host cell and many of them suppress host defense responses. Interestingly, certain symbiotic strains of rhizobia also possess such secretion systems. Rhizobia infect legume roots and induce root nodules, where the bacteria convert atmospheric nitrogen into ammonia. Here, we characterize the effector NopM of *Rhizobium* sp. strain NGR234. We demonstrate that NopM possesses E3 ubiquitin ligase activity, indicating that NopM can “tag" proteins with ubiquitin, and thus target them for proteasome-dependent degradation. Using a mutant approach, we demonstrate that enzymatically active NopM promotes establishment of symbiosis with *Lablab purpureus*, the host plant from which NGR234 was originally isolated. We further examine effects of NopM when directly expressed in eukaryotic cells and show that NopM interferes with specific signaling pathways. NopM expressed in the model plant *Nicotiana benthamiana* dampened generation of reactive oxygen species (ROS), which are formed in response to the bacterial flagellin peptide flg22. We suggest that NopM promotes nodule initiation by reducing the levels of harmful ROS during the infection process.

## Introduction

Type 3 effector proteins of pathogenic Gram-negative bacteria are transported into eukaryotic host cells through the bacterial type 3 secretion system (T3SS), which forms a needle-like pilus [1]–[3]. Various effectors from phytopathogenic bacteria act as virulence factors by suppressing activation of plant defense genes, i.e. they inhibit innate immunity triggered by highly conserved ubiquitous microbial elicitors (microbe-associated molecular patterns – MAMPs) such as flagellin, also called pattern-triggered immunity. On the other hand, plants can also possess resistance (R) proteins that mediate defense (effector-triggered immunity) by directly or indirectly recognizing specific type 3 effectors (avirulence factors). Hence, type 3 effectors of pathogenic bacteria can positively or negatively affect pathogenicity [1]–[3].

Interestingly, certain rhizobia also use type 3 effectors during symbiosis with host legumes [4], [5]. Rhizobia are nitrogen-fixing bacteria which establish a specific mutualistic endosymbiosis with legumes and certain species of the genus *Parasponia*. As a result of rhizobial infection, roots of host plants develop nodules, in which the bacteria differentiate into bacteroids. For the host's benefit, atmospheric nitrogen is then reduced to ammonia by the bacterial nitrogenase enzyme. During nodule formation, various signal molecules are exchanged between the two partners [6], [7]. Flavonoids released by host plants into the rhizosphere interact with rhizobial transcriptional regulators (NodD proteins). As a result, symbiotic genes involved in synthesis of bacterial nodulation signals (Nod factors) are activated. In certain rhizobial strains, such as *Rhizobium* sp. strain NGR234 [8], NodD-flavonoid interactions also result in stimulated expression of *ttsI*. This gene encodes a transcriptional activator, which controls expression of genes that have a conserved *cis*-element in their promoters, named *tts*-box. In NGR234 and a number of other strains, genes encoding a bacterial type 3 secretion system (T3SS) and corresponding type 3 effectors are regulated by TtsI [9], [10]. Mutant analysis revealed that type 3 effectors of NGR234 can play a role during symbiosis. Depending on the host plant, positive, negative or no effects on symbiosis have been reported [11]–[16].

One approach to study the function of bacterial effectors is to express them singly in eukaryotic cells. The type 3 effector proteins NopL and NopT (nodulation outer proteins L and T) of strain NGR234 have been characterized in this way. When expressed in tobacco and *Lotus japonicus*, NopL suppressed expression of defense genes [17]. NopL was multiply phosphorylated within eukaryotic cells and interfered with mitogen-activated protein (MAP) kinase signaling in yeast and tobacco cells [16]. Indeed, nodules of certain bean cultivars colonized by NGR234 mutated in *nopL* rapidly developed necrotic areas, indicating a lack of suppression of defense [16]. The protease NopT, another type 3 effector of NGR234 belonging to the YopT-AvrPphB effector family, influenced nodulation of host plants either positively or negatively [14], [15]. Accordingly, when transiently expressed in tobacco plants, proteolytically active NopT elicited a rapid hypersensitive reaction, suggesting that NopT action induced an R-protein mediated defense response in this non-host plant [14]. Similarly, resistance (R) genes (*Rj2* and *Rfg1*) of certain soybean cultivars are involved in host-specific nodulation and prevented establishment of symbiosis with specific strains in a T3SS-dependent manner [18].

The leucine-rich repeat (LRR) protein NopM (nodulation outer protein M) was first identified in *Sinorhizobium fredii* HH103 using a proteomic approach, in which secreted proteins from a T3SS-deficient mutant were compared to proteins from wild-type bacteria [19]. Homologous sequences exist in various rhizobial strains, namely *Rhizobium* sp. strain NGR234 (*nopM* formerly y4fR), *Bradyrhizobium japonicum* USDA110 (blr1904 and blr1676) and *B. elkanii* USDA 61 (*nopM*). During the course of the present study, T3SS-dependent secretion of NopM has been reported for strain NGR234 and a *nopM* deletion mutant induced fewer nodules on the host *Lablab purpureus* compared to the parent strain [15]. The *nopM* promoter activity depended on TtsI, which is predicted to bind to the conserved *tts* box (TB1) in the promoter region of *nopM* [10]. Based on sequence comparisons, rhizobial NopM proteins are predicted type 3 effectors belonging to the IpaH effector family with representatives in *Shigella flexneri* (such as IpaH9.8 and IpaH1.4) and *Salomonella enterica* (SspH1, SspH2, SlrP) [20]–[24]. The NopM sequence is also related to the YopM effector of *Yersinia pestis* [25]. Sequence similarities, albeit less related, also exist for non-characterized effectors from other bacteria, including the phytopathogens *Pseudomonas syringae* and *Ralstonia solanacearum* (e.g. HpX29 of *R. solanacearum* strain RS1000) [26].

IpaH family effectors are E3 ubiquitin ligases with a NEL (novel E3 ligase) domain. Enzymatic activity has been demonstrated for effectors from *S. flexneri* (such as IpaH9.8 and IpaH1.4) and *S. enterica* (SspH1, SspH2, SlrP) [20]–[24]. E3 ubiquitin ligases mediate transfer of ubiquitin from an E2 ubiquitin conjugating enzyme to a given target protein in eukaryotic cells, which is thereby marked for degradation. Ubiquitin-mediated proteasome-dependent protein degradation is conserved in eukaryotic cells. Ubiquitination itself requires three enzymatic components. First, an ubiquitin-activating enzyme (E1) forms a thioester bond between a catalytic cysteine and the carboxy terminal glycine residue of ubiquitin. The ubiquitin is then transferred to an ubiquitin-conjugating enzyme (E2). Finally, an E3 ubiquitin ligase facilitates the covalent conjugation of ubiquitin from an ubiquitin-loaded E2 to one or more lysine residues in a given protein substrate [27].

Bacterial E3 ubiquitin ligases delivered into host cells mimic the activities of host E3 ubiquitin ligases and ubiquitinate specific target proteins. For example, IpaH9.8 *of S. enterica* blocks the innate immune system of human cells by interfering with the nuclear factor κB (NF-κB) pathway. IpaH9.8 interacts with NEMO (NFκB essential modifier or IKKγ; an essential component of the multi-protein IKK (IκB kinase) complex) and the ubiquitin-binding adaptor protein ABIN-1. As a result, NEMO is polyubiquitinated and NF-κB activation is suppressed [28]. The SlrP effector of *S. enterica* targets thioredoxin and ERdj3, an endoplasmic reticulum luminal chaperone [29].

In this study, we characterize NopM of *Rhizobium* sp. strain NGR234. We demonstrate that NopM possesses E3 ubiquitin ligase activity. Our mutant analysis reveals that NopM acts as an E3 ubiquitin ligase during symbiosis with the host *L. purpureus*. NopM activity also inhibits mating pheromone signaling when expressed in yeast, and MAMP-triggered generation of reactive oxygen species (ROS) when expressed in *Nicotiana benthamiana* plants, while stimulating expression of MAMP-induced defense genes at the same time. We discuss our results in the light of the role of NopM in symbiosis.

## Results

### NopM possesses E3 ubiquitin ligase activity

**Figure 1:**
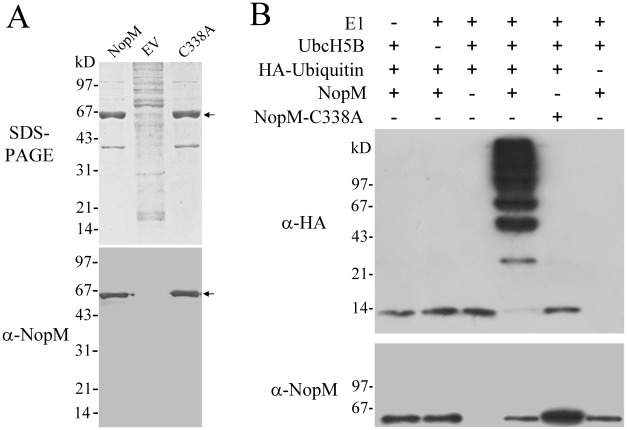
NopM possesses E3 ubiquitin ligase activity. (**A**) Purification and immunoblot analysis of His-tagged NopM and point-mutated His-tagged NopM-C338A (marked by an arrow). Proteins purified by nickel–nitrilotriacetic acid affinity chromatography were obtained from *E. coli* BL21 (DE3) harboring pET-*nopM* (lane NopM) and pET-*nopM*(C338A) (lane C338A), respectively. BL21 (DE3) with the empty vector pET28b was used as a control (lane EV). The SDS-PAGE gel (0.5 µg loaded proteins) was stained with Coomassie Brilliant Blue R-250 and the corresponding immunoblot with the rabbit serum raised against NopM was developed with 3, 3′-diamino-benzidine. (**B**) *In vitro* ubiquitination reactions with indicated purified proteins followed by immunoblot analysis with chemiluminescence reagents using anti-HA and anti-NopM antibodies. Ubiquitination reactions were performed in the presence or absence of HA-tagged ubiquitin, E1, UbcH5B, His-tagged NopM and His-tagged NopM-C338A for 1 h at 37°C.

The coding region of *nopM* was cloned into pET28b resulting in plasmid pET-*nopM*. A second plasmid, pET-*nopM*(C338A), was constructed in which the cysteine residue 338 of NopM was replaced by alanine. Residue C338 in the C-terminal NEL domain is predicted to be an essential catalytic residue required for the ubiquitin transfer [20]–[22]. *Escherichia coli* BL21 (DE3) harboring the constructed plasmids were induced with IPTG and extracted proteins were purified using nickel–nitrilotriacetic acid affinity chromatography. When analyzed by SDS-PAGE, a strong band with an apparent molecular mass of about 65 kD was detected, which corresponded to His-tagged NopM and NopM-C338A, respectively (calculated molecular weight of NopM ≈60.5 kD). This band was not seen, when proteins from *E. coli* BL21 (DE3) harboring the empty vector pET28b were purified in a similar way. Immunoblot analysis revealed that corresponding anti-NopM antibodies recognized His-tagged NopM and NopM-C338A proteins (Figure 1A).

His-tagged NopM and NopM-C338A purified from *E. coli* cells were then tested using an *in vitro* E3 ubiquitin ligase assay using HA-tagged ubiquitin as a substrate. After incubation, reaction mixtures were separated by SDS-PAGE and corresponding immunoblots were performed with anti-HA or anti-NopM antibodies. As shown in Figure 1B, when ubiquitination reactions were performed with His-NopM, anti-HA antibodies recognized a ladder of ubiquitinated proteins in the range of 24 to >200 kD. The size of proteins detected by the anti-HA antibodies (27 kD, 45 kD and 63 kD bands) were multiples of the size of HA-ubiquitin (9 kD), indicating formation of polyubiquitination chains. In contrast, reactions with NopM-C338A did not result in a ladder of polyubiquitinated proteins. Anti-NopM antibodies recognized a 65-kD protein band corresponding to His-tagged NopM and NopM-C338A, respectively. No additional bands of higher molecular weight were observed, indicating that NopM itself was not autoubiquitinated (Figure 1B). Taken together, these findings show that NopM is an E3 ubiquitin ligase and that the mutant protein NopM-C338A lacks this enzyme activity.

### Only enzymatically active NopM promotes nodulation of *Lablab purpureus*

**Figure 2:**
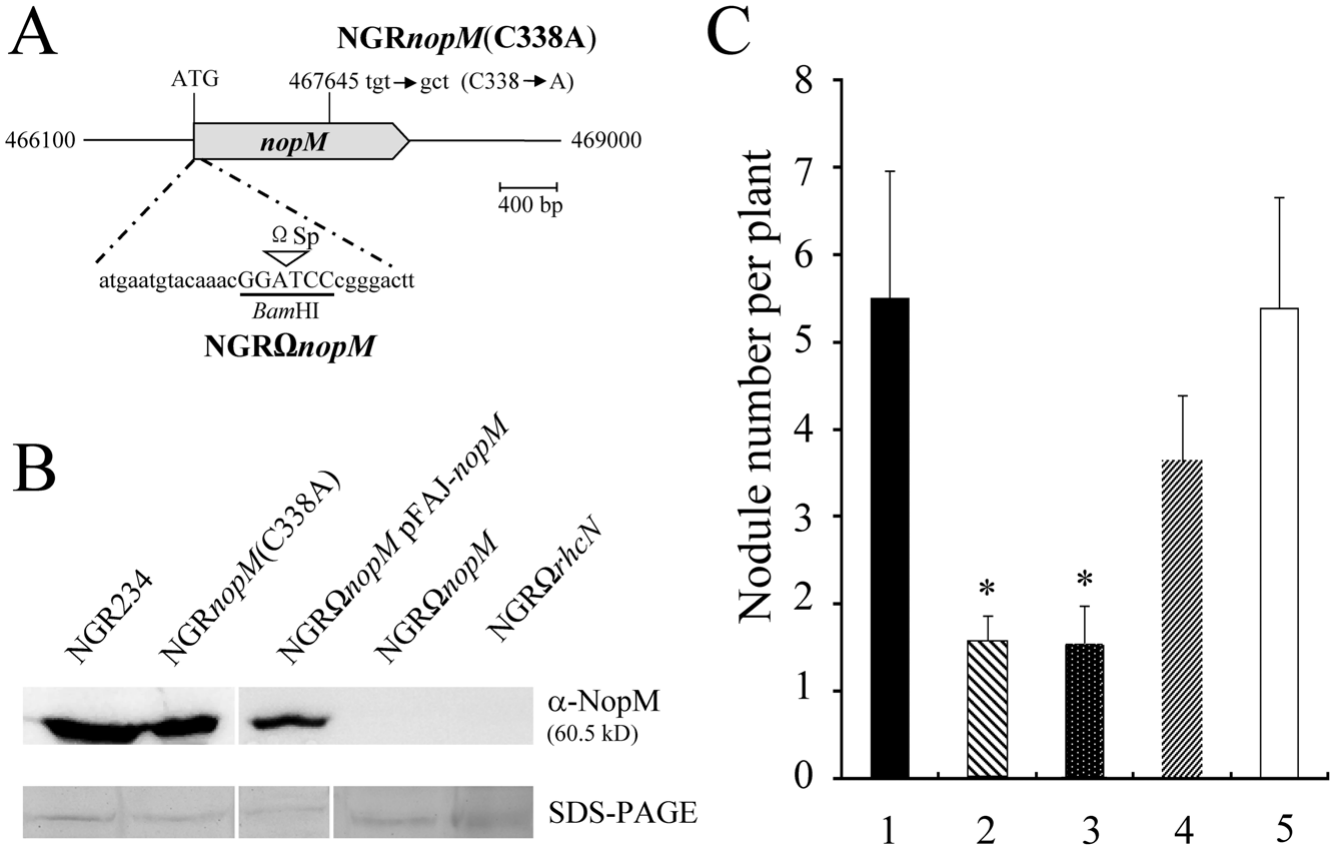
Characterization of the mutant strains NGRΩ*nopM* and NGR*nopM*(C338A). (**A**) Schematic representation of the knock-out mutant NGRΩ*nopM* (with an insertion of a 2-kb spectinomycin Ω interposon (Ωsp) at the constructed *Bam*HI restriction site) and of the point mutant NGR*nopM*(C338A) (cysteine 338 mutated to alanine; additional *Aor*51HI restriction site). (**B**) Immunoblot analysis of NopM of *Rhizobium* sp. NGR234 and indicated mutant derivatives. Immunoblots were performed with secreted proteins and the anti-NopM antibodies. As a loading control, secreted proteins in the molecular weight range of NopM were visualized on a parallel SDS-PAGE gel by silver staining (**C**) Symbiotic phenotype of *Rhizobium* sp. NGR234 (column 1), NGRΩ*nopM* (column 2), NGR*nopM*(C338A), (column 3), NGRΩ*nopM* carrying pFAJ-*nopM* (column 4) and NGR*nopM*(C338A) carrying pFAJ-*nopM* (column 5) on the host plant *L. purpureus* (cv. Chaojibiandou). In total, 70 plants (14 plants per strain) were inoculated. The numbers of nodules formed per plant were determined 35 days post inoculation. Data indicate means ± SE. Asterisks indicate significant reduced nodule formation as compared to the parent strain NGR234 (Kruskal-Wallis rank sum test; P<0.01).

Two mutant derivatives of *Rhizobium* sp. NGR234 were constructed to examine the function of NopM during symbiosis. A *nopM* knock-out mutant, called NGRΩ*nopM*, was generated, which contained an Ω spectinomycin interposon close to the ATG start codon. A point mutant, called NGR*nopM*(C338A), was constructed by using a corresponding DNA sequence encoding NopM-C338A (Figure 2A). Proteins from apigenin-induced culture supernatants were concentrated and used for immunoblots with the anti-NopM antibodies. NopM and NopM-C338A (ca. 60-kD bands) were detected in culture supernatants from the parent strain NGR234 and the NGR*nopM*(C338A) mutant, respectively. As expected, no bands were seen for the knock-out mutant NGRΩ*nopM*. Strain NGRΩ*rhcN*, a mutant lacking a functional T3SS served as a negative control [11]. NGRΩ*nopM* carrying the plasmid pFAJ-*nopM* (containing *nopM* including its promoter sequence) secreted NopM, indicating complementation by this plasmid (Figure 2B).

Nodulation phenotypes of the examined strains differed when the legume *L. purpureus* was inoculated. Figure 2C shows the results for a representative nodulation experiment. The parent strain NGR234 induced about 5–6 nodules per plant under the tested growth conditions. In contrast, NGRΩ*nopM* induced significantly fewer nodules (1–2 nodules per plant), indicating that NopM was required for optimal nodulation of this host plant. Plants inoculated with NGR*nopM*(C338A) showed a similar reduction in nodulation, indicating that the C338 residue is essential for the nodule-promoting effect of NopM. The symbiotic phenotype of NGRΩ*nopM* and NGR*nopM*(C338A) on *L. purpureus* could be complemented when plasmid pFAJ-*nopM* was introduced into these mutants. The nodule number was significantly increased and reached values comparable to those of the parent strain NGR234 (Figure 2C).

Nodulation tests were also performed with *Phaseolus vulgaris* (cv. Yudou No 1). Optimal nodulation of this plant with NGR234 required NopT, another type 3 effector of NGR234 [14]. Nodulation data with either NGRΩ*nopM* or NGR*nopM*(C338A) were similar to those obtained from the parent strain NGR234, however. Similarly, nodulation tests with the constructed mutants showed no obvious differences for *Flemingia congesta* (data not shown), although nodulation of this host plant is improved by a functional T3SS [12].

Taken together, the mutant analysis revealed that the symbiotic phenotype of the constructed *nopM* mutants depended on the tested host legume and that the positive effect of NopM on *L. purpureus* nodulation likely depended on its E3 ubiquitin ligase activity.

### Effects of NopM in yeast cells

**Figure 3:**
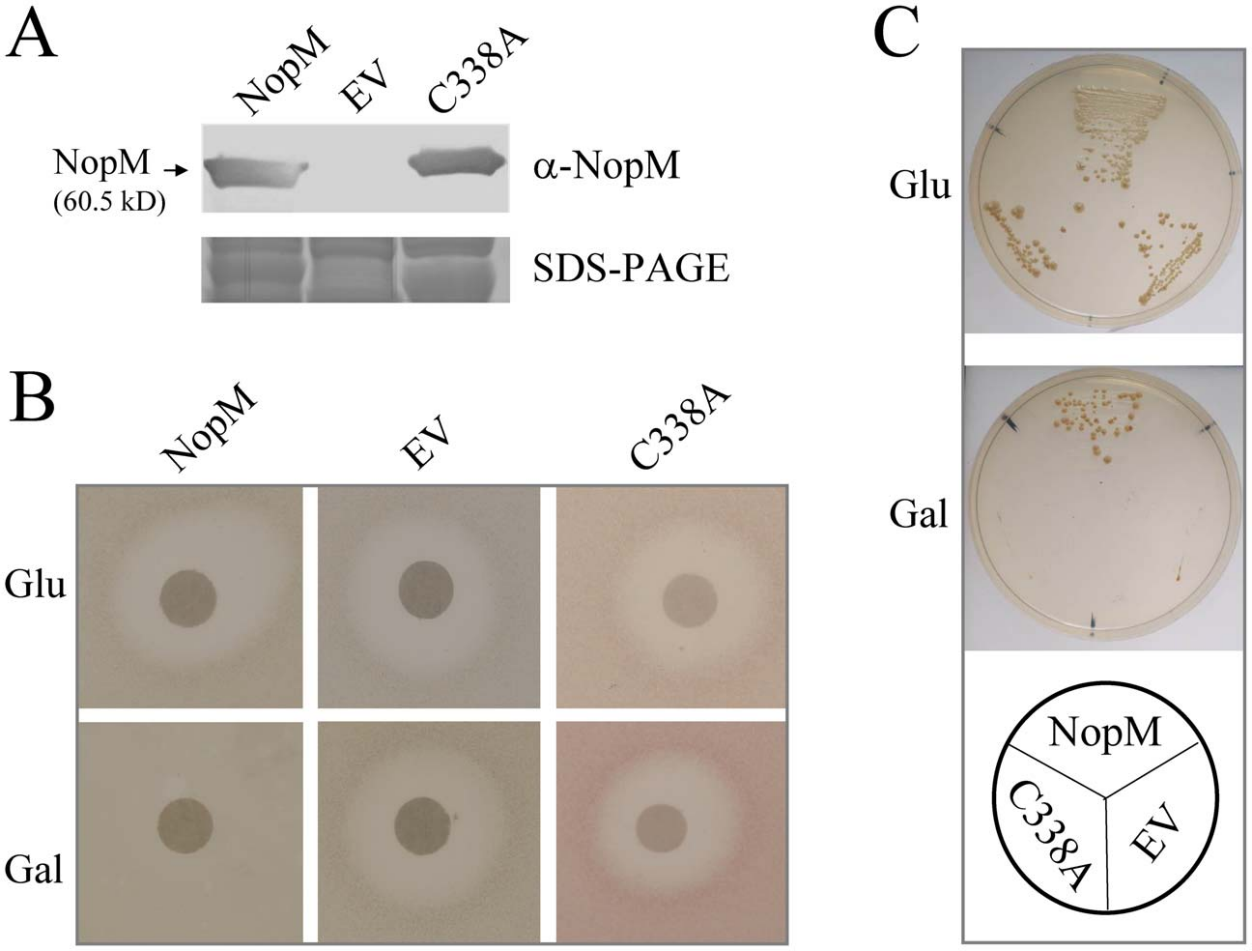
NopM inhibits mating pheromone signaling in yeast. (**A**) Immunoblot analysis of NopM and NopM-C338A isolated from yeast strain W303-1A carrying pESC-*nopM* (lane NopM) and pESC-*nopM*(C338A) (lane C338A), respectively. Proteins from W303-1A containing the empty vector pESC-leu (lane EV) were analyzed as a control. Extracted proteins (0.5 µg) were probed using the anti-NopM antibodies. A parallel SDS-PAGE gel was stained with Coomassie Brilliant Blue R-250 (**B**) Halo assay with glucose (Glu) or galactose (Gal) containing plates of yeast strain W303-1A harboring the plasmids pESC-*nopM*, the empty vector pESC-leu or pESC-*nopM*(C338A), respectively. A disk impregnated with 10 µg of α-factor was added to the center of the plate. (**C**) Growth of strain SY2227 (STE4 expression on SD/galactose plates) transformed with pESC-*nopM* (NopM expression on SD/galactose plates), pESC-*nopM*(C338A) (NopM-C338A expression on galactose plates) or the empty vector pESC-leu. Yeast cells were spread on SD medium plates containing either 2% (w/v) glucose (Glu) or 2% (w/v) galactose (Gal) without leucine.

When expressed in yeast, IpaH9.8 of *S. flexneri* blocked mating pheromone (α-factor) response signaling, a specific MAP kinase pathway [20]. We used the same type of assay to study the effect of NopM when expressed in yeast. The α-factor is perceived by a G protein-coupled receptor and activation of the signal cascade results in arrest of the cell cycle and transcription of mating genes. Accordingly, application of α-factor to the center of an agar plate of strain W303-1A (*MAT*a) results in a typical halo of growth inhibition [30]. The coding sequence of *nopM* and the point-mutated sequence encoding NopM-C338A were cloned into the expression vector pESC-leu, which has a galactose-inducible promoter (GAL1). W303-1A cells carrying the resulting plasmids (pESC-*nopM* and pESC-*nopM*(C338A), respectively) expressed NopM and NopM-C338A on galactose plates: An immunoblot with anti-NopM antibodies exhibited a band corresponding to the predicted size of NopM (60.5 kD), which was absent in cells transformed with the empty vector pESC-leu (Figure 3A). Upon exposure to α-factor, yeast cells expressing *nopM* under the GAL1 promoter failed to form a halo, indicating that NopM interfered with the mating pheromone signaling pathway. Using the same assay, NopM-C338A did not inhibit mating pheromone response signaling (Figure 3B).

A similar growth inhibition assay was performed with yeast strain SY2227, which expresses the G protein β-subunit STE4 when the fungus is grown on galactose-containing media. Overproduction of STE4 activates the mating pheromone signaling pathway and therefore causes cell growth arrest in the absence of α-factor [31]. Figure 3C shows the growth phenotype of this strain transformed with pESC-*nopM*, pESC-*nopM*(C338A) or the empty vector pESC-leu. Yeast transformed with pESC-*nopM* showed normal growth on SD/galactose plates. In contrast, cells transformed with pESC-*nopM*(C338A) or pESC-leu poorly grew on SD/galactose (Figure 3C). Hence, NopM, but not NopM-C338A, inhibited STE4-induced mating pheromone signaling.

### Effects of NopM in *Nicotiana benthamiana*

**Figure 4:**
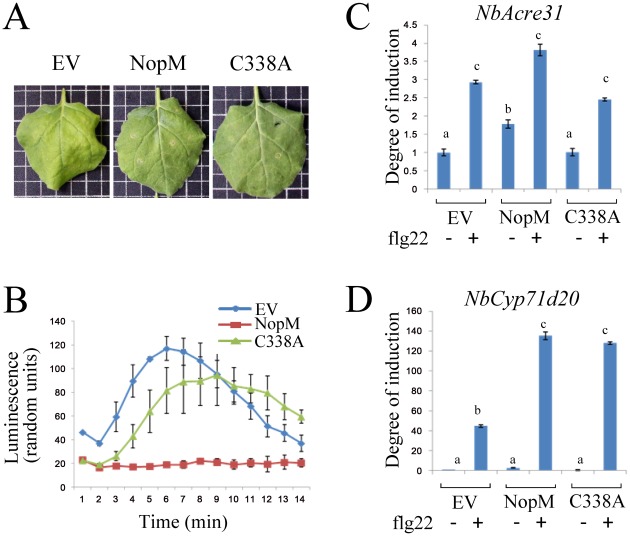
Effects of NopM and NopM-C338A on *N. benthamina* cells. Leaves were infiltrated *with A. tumefaciens* strain GV3101 carrying pCAMBIA-*nopM*, pCAMBIA-*nopM*(C338A) or the empty vector pCAMBIA-T. (**A**) Leaves were photographed two days post infiltration. (**B**) ROS production in transformed leaf disks. Two days post infiltration, leaf disks were treated with 1 µM of the elicitor flg22. (**C**) Levels of *NbAcre31* transcripts. Two days after infiltration, leaf disks were treated with 1 µM flg22 in BSA/NaCl solution. Control leaves were mock treated with BSA/NaCl solution in the absence of flg22. RNA was isolated from tissue harvested 30 min later. qRT-PCR was performed with three biological and three technical replicates. Data (means ± SE) indicate the degree of induction as compared to leaves infiltrated with *A. tumefaciens* carrying the empty vector. Different letters above columns indicate statistically different transcript levels (**D**) Levels of *NbCyp71d20* transcripts. Expression data were obtained as for *NbAcre31*.

To investigate effects of NopM within plant cells, NopM and NopM-C338A were transiently expressed in *N. benthamiana*. DNA encoding NopM or NopM-C338A was cloned into the binary vector pCAMBIA-T, which contains a 35S cauliflower mosaic virus 35S promoter. *Agrobacterium tumefaciens* cells carrying these vectors were then used for infiltration of *N. benthamiana* leaves. Immunoblot analysis with anti-NopM antibodies revealed the presence of NopM and NopM-C338A proteins in transformed tissue (Figure S1 in Text S1, panel A). Leaves expressing NopM (2 days after infiltration) showed no hypersensitive reaction (Figure 4A). Trypan blue based cell death staining of leaves (5 days after infiltration) showed that neither NopM nor NopM-C338A caused visible changes as compared to leaf tissue transformed with the empty vector (Figure S1 in Text S1, panel B). The *P. syringae* pv. *tomato* DC3000 effector HopQ1, which is known to induce a hypersensitive reaction in *N. benthamiana* [32], was used as a positive control. As expected, the HopQ1 expressing tissue was necrotic and strongly stained by trypan blue.

Transient generation of reactive oxygen species (ROS) induced by MAMPs is a rapid signaling response, which depends on Rboh enzymes (respiratory burst oxidase homologs) and is activated by calcium-dependent protein kinases (CDPKs) [33], [34]. When challenged with flg22, a conserved, 22-amino acid motif of the bacterial MAMP flagellin [1], *N. benthamiana* leaf disks respond with a ROS burst, which can be measured with luminol and horseradish peroxidase [35]. An example for such an experiment is shown in Figure 4B. Interestingly, the flg22-induced ROS burst was nearly completely abolished in leaf disks expressing NopM (statistical analysis of all data from 4 independent time series; significant differences as compared to controls transformed with the empty vector; one-way ANOVA, *P* = 0.002). In disks expressing NopM-C338A, however, the ROS burst in response to flg22 was similar to empty vector controls (p = 0.24). Accordingly, differences between disks expressing NopM and NopM-C338A were significant (one-way ANOVA, *P* = 0.008), suggesting that ROS suppression depends on the ubiquitin E3 ligase activity of NopM (Figure 4B).

Transcript levels of the flg22-responsive defense genes *NbAcre31* (encoding a putative calcium-binding protein) and *NbCyp71d20* (encoding a putative cytochrome P450) are upregulated in response to flg22 [36]. Quantitative reverse transcription (qRT)-PCR was used to examine the effect of NopM on expression of these genes (Figure 4C and D). In the absence of flg22, NopM expression resulted in slightly elevated transcript levels of *NbAcre31* (one-way ANOVA, p = 0.03), but not of *NbCyp71d20* (p = 0.08). In contrast, cells expressing NopM-C338A neither showed increased transcript levels of *NbAcre31* nor of *NbCyp71d20*. As expected, leaf tissue challenged with flg22 showed stimulated expression, particularly for *NbCyp71d20*. When compared to empty vector controls, effects of the flg22 treatment on *NbCyp71d20* activation were significantly stronger in either NopM or NopM-C338A expressing tissues (Figure 4D). Thus, NopM promoted flg22-induced *NbCyp71d20* expression independently of its E3 ubiquitin ligase activity.

The flg22-induced expression of *NbAcre31* and *NbCyp71d20* depends on the MAP kinase SIPK (salicylic acid-induced protein kinase) [37]. The amounts of active MAP kinases in *N. benthamiana* were visualized on immunoblots with anti-p42/44-phospho-ERK antibodies, which recognize activated SIPK and WIPK (wound-induced protein kinase). Expression of NopM in *N. benthamiana* did not result in MAP kinase activation. A treatment of leaves with flg22 for 15 min caused MAP kinase activation in control plants transformed with the empty vector as well as in NopM or NopM-C338A expressing plants, indicating that NopM did not block flg22-induced MAP kinase signaling (Figure S1 in Text S1, panel C). In the presence of NopM and flg22, activation of SIPK might be slightly stronger, but a more quantitative approach would be required to reveal small changes in MAP kinase activation.

## Discussion

We show in this study that the LRR protein NopM of the rhizobial symbiont NGR234 is an E3 ubiquitin ligase belonging to the IpaH effector family. Effectors of this family are also known as NEL (novel E3 ligase) domain effectors. These enzymes are structurally unrelated to other bacterial E3 ubiquitin ligases, which have a HECT or RING/U-box domain [38]. The NEL domain in the C-terminal region of NopM was functional in NopM, whereas the NopM-C338 mutant protein was inactive. The C338 residue of NopM likely acts as a nucleophile, forming a thioester bond with ubiquitin. Enzymatic activities of NEL domain effectors have been only reported for the human pathogens *S. flexneri* and *S. enterica* [20]–[24]. Thus, NopM represents a first studied example for a NEL domain effector delivered into plant cells.

Inoculation tests with the constructed NGR234 mutants, NGRΩ*nopM* and NGR*nopM*(C338A), showed reduced nodulation on *L. purpureus*, indicating the importance of the C338 residue during establishment of symbiosis. We suggest that NopM functions as an E3 ubiquitin ligase during the infection process and that ubiquitination of one or more host proteins helps to promote nodulation on *L. purpureus*. In some other host plants, however, effects of NopM on nodulation were either not observed or even negative ([15] and this study). It is tempting to speculate that NopM function reflects an evolutionary adaptation to protein substrates of specific hosts. Indeed, NGR234 has been originally isolated from *L. purpureus* [39] whose nodulation is promoted by NopM. Negative effects of NopM on nodulation in other legumes are potentially related to specific R-proteins of the host plant as shown for T3SS-dependent nodulation of certain soybean cultivars [18].

Similar to many Gram-negative pathogenic bacteria, rhizobial T3SSs are believed to deliver type 3 effector proteins into host cells. Translocation of rhizobial type 3 effectors into legume cells has been questioned [40]. However, recent evidence has provided strong support, based on transfer of adenylate cyclase fused to rhizobial effectors [41], [42]. Our findings indicate that NopM, but not NopM-C338A, promoted nodule formation in *L. purpureus*. In fact, delivery into host cells is a prerequisite for bacterial E3 ubiquitin ligases, as they function in combination with ubiquitin and E1/E2 enzymes, which are present only in the eukaryotic cell.

Yeast is a model to investigate effects of type 3 effector proteins in eukaryotic cells [43]. The IpaH9.8 effector of *S. flexneri* blocked the mating pheromone response signaling in yeast and ubiquitinated Ste7, the mitogen-activated protein kinase kinase of this pathway [20], [21]. Our data point to a similar activity of NopM in yeast and these findings prompted us to investigate whether NopM can interfere with MAP kinase signaling in plants. Interestingly, *N. benthamiana* plants expressing NopM did not show suppression of flg22-induced MAP kinase signaling. Instead, the flg22-associated ROS burst was nearly completely abolished in plants expressing NopM. These findings are consistent with the concept that early flagellin signaling is split in two separate signaling branches, one leading to MAP kinase activation and the other to calcium-dependent protein kinase (CDPK) mediated ROS production [34], [37].

Suppression of flg22-triggered ROS in *N. benthamiana* depended on the C338 residue of NopM, suggesting ubiquitination of a MAMP signaling component. The E3 ubiquitin ligase activity of NopM is reminiscent of the virulence function of AvrPtoB, a type 3 effector of *P. syringae* with a functional E3 ubiquitin ligase RING/U-box E3 domain [44]. In *Arabidopsis thaliana*, AvrPtoB targets proteins such as the flagellin receptor complex FLS2-BAK1 [45] and the chitin receptor kinase CERK1 [46]. In contrast to ROS suppression, expression of either NopM or NopM-C338A in *N. benthamiana* promoted flg22-triggered accumulation of *NbCyp71d20* transcripts. Hence, ubiquitination activity of NopM was not essential to induce this effect. We suggest that an interaction between NopM and a *N. benthamiana* protein is sufficient to cause a partial deregulation of immune signaling in flg22-challenged tissue.

Taken together, we provide genetic and biochemical evidence that NopM is a type 3 effector with a functional NEL domain. Inoculation tests with the constructed point mutant NGR*nopM*(C338A) suggest that the E3 ubiquitin ligase activity of NopM is required for optimal nodulation of the host plant *L. purpureus*. When expressed in *N. benthamiana*, NopM suppresses the flg22-elicited ROS burst, suggesting that NopM blocks ROS-associated defense responses. Future work is required to test whether NopM can also suppress ROS formation in legume roots. Indeed, ROS generation could be detrimental during the rhizobial infection process [5], [47] and it is tempting to speculate that NopM keeps ROS generation in *L. pupurpureus* infection threads below a harmful threshold level.

## Materials and Methods

### Strains, plasmids and primers

Bacterial strains and plasmids used for this study are listed in Table S1 of Text S1. Plasmids were constructed according to standard methods and corresponding PCR primers are listed in Table S2 of Text S1.

### Expression of NopM and NopM-C338A in *E. coli*

The sequence encoding NopM of *Rhizobium* (*Sinorhizobium fredii*) sp. NGR234 (accession number AAB91674) was cloned into the pET28b vector, resulting in plasmid pET-*nopM*. PCR-based site-directed mutagenesis was used to mutate the cysteine 338 (TGT codon) of NopM into alanine (GCT codon) and the resulting plasmid was named pET-*nopM*(C338A). The plasmids were then transformed into *E. coli* BL21 (DE3) cells. The His-tagged NopM and NopM-C338A proteins were purified from isopropyl-β-D-thiogalactopyranoside (IPTG) induced cultures by nickel affinity chromatography with Ni-NTA resin beads (Qiagen, Hilden, Germany). For immunization of a New Zealand rabbit, Ni-NTA purified His-tagged NopM was separated by SDS-PAGE and gel bands containing NopM were cut from the gel.

### Ubiquitination test

Purified ubiquitin-activating enzyme from human (E1), UbcH5B (E2), and HA-ubiquitin were purchased from Boston Biochem (Cambridge, MA, USA). His-tagged NopM and NopM-C338A from *E. coli* cultures grown at 27°C for 12 h were purified by nickel affinity chromatography according to the manufacturer's recommendations under non-denaturing conditions (Qiagen, Germany). Ubiquitination assays were performed in a 40-µl volume containing the reaction buffer (25 mM Tris HCl (pH 7.5), 50 mM NaCl, 5 mM ATP, 10 mM MgCl_2_, 0.1 mM DTT), 2 µg HA-ubiquitin, 0.5 µg of E1, and 2 µg of UbcH5B in the presence or absence of 1 µg of His-tagged NopM, or NopM-C338A, respectively. Reactions were incubated at 37°C for 1 h and stopped by addition of an equal volume of Laemmli sample buffer (62.5 mM Tris HCl (pH 6.8), 10% (v/v) glycerol, 2% (w/v) SDS, 0.005% (w/v) bromophenol blue) containing 100 mM DTT. Reaction mixtures were separated by SDS-PAGE, transferred onto a nitrocellulose membrane, and probed with specific antibodies (anti-NopM antibodies at 1∶10 000 dilution; anti-HA antibodies from (Abcam, England) at 1∶ 4 000 dilution). Immunoblots were developed with enhanced chemiluminescence reagents (GE Healthcare).

### Construction of NGRΩ*nopM* and NGR*nopM*(C338A)

For construction of the mutant NGRΩ*nopM*, a 2.5-kp fragment containing *nopM* was cloned into pBluescript II KS(+), generating pSK-*nopM*2500. PCR-based site-directed mutagenesis was used to generate a *Bam*HI restriction site close to the ATG codon of *nopM*. A spectinomycin-resistant (Sp^r^) Ω interposon was excised from pHP45 [48] with *Bam*HI and ligated into the *Bam*HI site, generating pSK-*nopM*Ω. The construct was then cloned into the suicide vector pJQ200SK [49]. The resulting plasmid (pJQ-*nopM*Ω) was mobilized from *E. coli* DH5α into *Rhizobium* sp. NGR234 by triparental mating using the pRK2013 helper plasmid [50]. Gene replacement was forced by selecting for the resistance of the Ω interposon marker (Sp^r^) and for growth on 5% (w/v) sucrose. The obtained mutant NGRΩ*nopM* was confirmed by Southern blot analysis using the DIG DNA labeling and detection kit as specified by the supplier (Roche, Basel, Switzerland).

For construction of NGR*nopM*(C338A), plasmid pSK-*nopM*2500 was mutated by a PCR-based site-directed mutagenesis approach, thereby creating the restriction site *Aor*51HI. The insert of this plasmid (named pSK-*nopM*(C338A)) with DNA encoding NopM-C338A was then cloned into the suicide vector pJQ200SK, resulting in plasmid pJQ-*nopM*(C337A). After conjugation, *Rhizobium* sp. NGR234 bacteria were first cultivated on agar plates containing gentamycin and rifampin and then on plates containing rifampin and 5% (w/v) sucrose. Genomic DNA from candidate colonies served as a template for a PCR (primers 11 and 12; Table S2 in Text S1). The amplicon from the mutant NGR*nopM*(C338A) was completely cleaved by *Aor*51HI into two smaller fragments.

For complementation analysis of the constructed mutants, a 2081-bp fragment containing the coding region and promoter sequence of *nopM* was cloned into pFAJ1702 [51]. The obtained plasmid (pFAJ-*nopM*) was then mobilized into *Rhizobium* sp. NGR234 and selection was performed on agar plates containing tetracycline.

### Isolation of secreted proteins

Secreted proteins from culture supernatants from *Rhizobium* sp. strains NGR234 (parent strain), NGRΩ*nopM* (this study), NGR*nopM*(C338A) (this study), NGRΩ*nopM* carrying pFAJ-*nopM* (this study) and NGRΩ*rhcN* [11] were isolated according to a previously described procedure [12], [52]. Briefly, cultures (RMS medium) were induced with 1 µM apigenin and cultivated at 27°C on a rotary shaker for 40 h. Proteins from culture supernatants were precipitated by addition of TCA (10%, w/v) and incubation over night at 4°C. After centrifugation (10 000× g, 4°C, 30 min), precipitates were washed twice with 5 ml of cold 80% acetone and resuspended in 100 µl of rehydration buffer (8 M urea, 2% w/v CHAPS, 0.01% w/v bromophenol blue). Secreted proteins (corresponding to 100 ml of cell culture) were subjected to immunoblot analysis with antiserum against NopM (1∶5 000 dilution) followed by staining with chemiluminescence reagents (Thermo Scientific, Waltham, MA USA).

### Nodulation tests

Nodulation tests were performed in plastic jars using the host plants *Lablab purpureus* cv. Chaojibiandou, *Phaseolus vulgaris* cv. Yudou No 1, and *Flemingia congesta*. Seeds were surface sterilized and germinated on agar plates, and plantlets were transferred to 300-mL plastic jar units linked with a cotton wick (a mixture of vermiculite and expanded clay in the upper vessel; nitrogen-free nutrient solution in the lower vessel). Plants (1 plant per jar) were inoculated with 10^9^ bacteria (strain NGR234 and mutant derivatives; see Table S1 in Text S1). Plants were cultivated at 26±2°C in a temperature-controlled greenhouse. The nodulation test results were statistically analyzed with the Kruskal-Wallis rank sum test, which is suitable for unequal replications. A *P*-value of ≤0.01 was considered as significant. All data are presented as means ± SE (standard error).

### Expression of NopM and NopM-C338A in yeast and halo assay with α-factor

Standard media and techniques were used for transformation, maintenance, and growth of *Saccharomyces cerevisiae* [53]. Strains (haploid strain W303-1A (*MAT*a) strain SY2227) and constructed plasmids encoding NopM or NopM-C338A are listed in Table S1 of Text S1. For immunoblot analysis, yeast cells were cultured at 30°C in liquid SD/-Leu medium (Clontech) supplemented with 2% galactose. Membranes were incubated with anti-NopM antibodies at a 1∶5 000 dilution and blots were developed with 3, 3′-diamino-benzidine (Boster, Wuhan, China). The halo assay with the mating pheromone was performed by placing a filter disk impregnated with 8 µg of the mating pheromone α-factor (Sigma-Aldrich; dissolved in 8 µl H_2_O) to the center of each agar plate. The plates were sealed, incubated at 27°C for 1 week and then photographed.

### Expression of NopM and NopM-C338A in *N. benthamiana*

Plasmids (pCAMBIA-*nopM*, pCAMBIA-*nopM*(C338A), the empty vector pCAMBIA-T and pGWB417-HopQ1-myc; see Table S1 in Text S1) were transformed into chemically competent *Agrobacterium tumefaciens* strain GV3101 by heat shock. Leaves from 4-week old *Nicotiana benthamiana* plants were infiltrated with bacteria (OD_600_ = 0.5) re-suspended in infiltration buffer (10 mM MgCl_2_, 10 mM MES pH 5.6). Expression of NopM was detected by immune blot analysis with anti-NopM antibodies at a 1∶1000 dilution. Blots were developed with CDPstar reagents (New England Biolabs). Staining of *N. benthamiana* leaves was performed with trypan blue as described previously [54].

### Measurement of reactive oxygen species (ROS) generation

Leaf discs (0.38 cm^2^) were floated on water overnight and ROS released by the leaf tissue were measured using a chemiluminescent assay [35]. The water was replaced with 500 µl of an aqueous solution containing 20 µM luminol (Sigma-Aldrich) and 1 µg of horseradish peroxidase (Fluka, Buchs, Switzerland). ROS was elicited with 1 µM flg22 peptide (QRLSTGSRINSAKDDAAGLQIA) in all experiments. Mock treatments without flg22 were performed with the BSA/NaCl solution (1% w/v BSA, 1% w/v mM NaCl) used to solubilize flg22. Luminescence was measured over a time period of 30 min using a luminometer (MicroLumat LB96P; EG&G Berthold). Data from 12 leaf disks derived from 4 independent infiltrations were statistically analyzed by one-way ANOVA considering *P*≤0.05 as significantly different.

### MAP kinase activation assay

Two days post *A. tumefaciens* transformation, *N. benthamiana* leaves transiently expressing NopM, NopM-C338A and empty vector controls were infiltrated with 1 µM flg22 peptide or mock-treated with BSA/NaCl for 15 min. Leaf discs (50 mg) were then frozen in liquid nitrogen and proteins were extracted in 100 µl extraction buffer (50 mM Tris-HCl pH 7.5, 150 nM NaCl, protease inhibitor cocktail from Sigma-Aldrich) for 30 min at 4°C. Subsequently 100 µl Lämmli loading buffer (2×) was added to each sample. Samples were subjected to immunoblot analysis using the anti-p42/44-phospho-ERK antibody (Sigma-Aldrich). Blots were developed using CDP-star technology (NEB).

### Quantitative reverse transcription (qRT)-PCR

One day post infiltration, *N. benthamiana* leaf discs expressing NopM, NopM-C338A or the empty vector (EV) control were collected, and then floated overnight in water. Leaf discs were subsequently treated with 1 µM flg22 or mock-treated with BSA/NaCl solution for 30 min and then frozen in liquid nitrogen. Total RNA was extracted using the NucleoSpin RNA Plant extraction kit (Machery-Nagel). The absence of genomic DNA was checked by PCR amplification of the housekeeping *NbEF1α* gene by using 1 µg of RNA (*NbEF1α* amplification crosses an exon/intron boundary). For analysis of gene expression, first-strand cDNA was synthesized from 1 µg of RNA using AMV reverse transcriptase (Promega) and an oligo (dT) primer (Microsynth), according to the manufacturer's instructions. For quantitative PCR, 5 µl of a 1/100 µl dilution of cDNA were combined with SYBR master mix. PCRs were performed in triplicates with the 7500 Real Time PCR system (Applied Biosystems). Data were collected and analyzed with the respective ABI analyzing program. The *NbEF1α* RNA was analyzed as an internal control and used to normalize the values for transcript abundance. All samples were related to the empty vector (EV) control. Primers for the genes *NbCyp71D20*, *NbAcre31* and *NbEF1α* are listed in Table S2 of Text S1. Data derived from three biological repeats were statistically analyzed by ANOVA (one-way ANOVA) considering *P*≤0.05 as significantly different.

### Accession number of NopM

NopM of *Rhizobium* (*Sinorhizobium fredii*) strain NGR234: AAB91674

## Supporting Information

Text S1 Text S1 contains Table S1 (Strains and plasmids used in this study), Table S2 (Primers used in this study) and Figure S1 (Analysis of *N. benthamiana* expressing NopM and NopM-C338A by immunoblot analysis, trypan blue based cell death staining and MAP kinase activation in response to flg22). Text S1 also contains references cited in Table S1 and an explanatory legend to Figure S1.
(PDF)
