# Supplementary material for: Functional Analysis of NopM, a Novel E3 Ubiquitin Ligase (NEL) Domain Effector of Rhizobium sp. Strain NGR234
Source: PLoS Pathog. 2012 May 17;8(5):e1002707. doi: 10.1371/journal.ppat.1002707 (PMC3355095; doi:10.1371/journal.ppat.1002707)
Supplement: Text S1 — contains Table S1 (Strains and plasmids used in this study), Table S2 (Primers used in this study) and Figure S1 (Analysis of N. benthamiana expressing NopM and NopM-C338A by immunoblot analysis, trypan blue based cell death staining and MAP kinase activation in response to flg22). Text S1 also contains references cited in Table S1 and an explanatory legend to Figure S1. (PDF) [file ppat.1002707.s001.pdf]

SUPPORTING INFORMATION FOR:

Da-Wei Xin, Sha Liao, Zhi-Ping Xie, Dagmar R. Hann, Lea Steinle, Thomas Boller, and Christian Staehelin (2012). Functional analysis of NopM, a novel E3 ubiquitin ligase (NEL) domain effector of *Rhizobium* sp. strain NGR234.

This file contains: Table S1, Table S2 and Figure S1

Table S1. Strains and plasmids used in this study

Strain or plasmid	Relevant characteristics	Reference
Escherichia coli		
DH5α	*supE44* Δ*lacU169* (ϕ*80lacZ*Δ*M15*) *hsdR17 recA1 endA1 gyrA96 thi-1 relA1*	GIBCO BRL, Bethesda, MD USA
BL21 (DE3)	F^- *ompT* $hsdS_B$ (r_B^- m_B^-) *gal dcm* (DE3)	Novagen (Merck Chemicals, Darmstadt, Germany)
Rhizobium sp.		
NGR234	*Rhizobium (Sinorhizobium, Ensifer)* sp. strain NGR234 isolated from *Lablab purpureus* (Rif^r derivative)	Trinick, 1980
NGRΩ*rhcN*	NGR234 derivative containing an Ωsp interposon in *rhcN* (Rif^r Sp^r)	Viprey *et al.,* 1998
NGRΩ*nopM*	NGR234 deletion mutant containing an Ωsp interposon in *nopM* (formerly y4fR) (Rif^r, Sp^r) (see Fig 2A)	This work

NGR*nopM*(C338A)	NGR234 with a point mutation in *nopM* (Rif^r) (see Fig 2A)	This work
Saccharomyces cerevisiae		
W303-1A	*MAT*a *ade2-1 can1-100 his3-11,15 leu2-3,112 rad5-535 trp1-1 ura3-1*	Wallis *et al.*, 1989
SY2227	*MATa ade1-1 leu2-2,113 trp1 ura3-52 bar1 HIS3::pFUS1::HIS3 mfa2-Δ1::FUS1-lacZ rad16::pGAL1::STE4*	Edwards *et al.*, 1997
Agrobacterium tumefaciens		
GV3101	pSOUP (Rif^r, Gm^r)	Hellens *et al.,* 2000
Plasmids		
pBluescript II SK (+)	High copy number ColE1-based phagemid (Amp^r)	Stratagene (Agilent Technologies, Shanghai, China)
pSK-*nopM*2500	A 2.5-kb fragment containing the promoter and coding region of *nopM* cloned into pBluescript II SK (+) using primers 5 and 6 and genomic DNA of NGR234 as template (Amp^r)	This work
pSK-*nopM*2500(*Bam*HI)	pSK-*nopM*2500 derivative obtained by site-directed mutagenesis with primers 7 and 8, generating a *Bam*HI restriction site	This work
pSK-*nopM*(C338A)	pSK-n*opM*2500 derivative obtained by site directed mutagenesis with primers 3 and 4; contains a created *Aor*51HI restriction site and a C to A point mutation in residue 338 of NopM	This work
pET28b	Expression vector based on pBR322 (Kan^r)	Novagen (Merck Chemicals, Darmstadt,

		Germany)
pET-*nopM*	A 1.64-kb fragment of the *nopM* coding region cloned into pET28b using primers 1 and 2 with pSK-*nopM2500* as template (Kan^r)	This work
pET-*nopM*(C338A)	A 1.64-kb fragment containing the sequence encoding NopM-C338A cloned into pET28b using primers 1 and 2 with pSK-*nopM*(C338A) as template (Kan^r)	This work
pHP45	Vector containing an Ω interposon (Amp^r, Sp^r)	Prentki and Krisch, 1984
pSK-*nopM*Ω	pSK-*nopM*2500(*Bam*HI) derivative containing a spectinomycin Ω cassette inserted into the *Bam*HI site (Amp^r)	This work
pJQ200SK	Suicide vector used for directed mutagenesis (Gm^r)	Quandt and Hynes, 1993
pJQ-*nopM*Ω	A 3.64-kb *Kpn*I-*Xba*I fragment excised from pSK-*nopM*Ω and cloned into pJQ200SK (Sp^r, Gm^r)	This work
pJQ-*nopM*(C338A)	pJQ200SK derivative carrying a 1.64-kb *Sac*I-*Xho*I fragment excised from pSK-*nopM*(C338A) (Gm^r)	This work
pRK2013	Tra$^+$ helper plasmid for mobilisation (Kan^r)	Figurski and Helinski, 1979
pFAJ1702	Gene expression vector (RK2 derivative) with symbiotic plasmid stability loci from *Rhizobium* sp. NGR234 (Tc^r)	Dombrecht *et al.*, 2001
pFAJ-*nopM*	A 2081-bp fragment containing the coding region and promoter sequence of *nopM*; PCR-cloned into pFAJ1702 with primers 13 and 14 using genomic DNA of *Rhizobium* sp. NGR234 as template	This work
pESC-leu	Yeast protein expression vector with a galactose-inducible promoter (GAL1), and a *LEU* gene as selective marker	Stratagene (Agilent Technologies,

	(Amp^r)	Shanghai, China)
pESC-*nopM*	A 1.64-kb fragment containing the coding region of *nopM* cloned into pESC-leu using primers 21 and 22 with pSK-*nopM*2500 as template (Amp^r)	This work
pESC-*nopM*(C338A)	A 1.64-kb fragment containing the sequence encoding NopM-C338A cloned into pESC-leu using primers 21 and 22 with pSK-*nopM*(C338A) as template (Amp^r)	This work
pRT104	Vector containing the cauliflower mosaic virus 35S promoter and a poly-(A) signal (Amp^r)	Töpfer *et al.*, 1987
pCAMBIA-T	pCAMBIA1305 derivative carrying a *Hind*III- *Sac*I fragment containing the cauliflower mosaic virus 35S promoter and a poly-(A) signal derived from pRT104 (Kan^r)	This work
pCAMBIA-*nopM*	pCAMBIA-T derivative containing the coding region of *nopM* amplified with primers 9 and 10 using pSK-*nopM*2500 as template (Kan^r)	This work
pCAMBIA-*nopM*(C338A)	pCAMBIA-T derivative encoding NopM-C338A; DNA amplified with primers 9 and 10 using pSK-*nopM*(C338A) as template (Kan^r)	This work
pGWB417	Gateway binary vector with the cauliflower mosaic virus 35S promoter and a C-terminal 4xMyc tag; accession number AB294441 (Sp^r)	Nakagawa *et al.,* 2007
pGWB417-HopQ1-myc	pGWB417 derivative containing the coding region of *HopQ1* of *Pseudomonas syringae* pv. *tomato* DC3000 (accession number AAO54411) (Sp^r)	This work

Amp^r, Gm^r, Km^r, Rif^r, Sp^r, Tc^r – resistance to ampicillin, gentamycin, kanamycin, rifampin, spectinomycin, and tetracycline, respectively.

References cited in Table S1

1. Bradford MM (1976) A rapid and sensitive method for quantitation of microgram quantities of protein utilizing the principle of protein-dye-binding. Anal Biochem 72:248-254.
2. Dombrecht B, Vanderleyden J, Michiels J (2001) Stable RK2-derived cloning vectors for the analysis of gene expression and gene function in gram-negative bacteria. Mol Plant Microbe Interact 14: 426-430.
3. Edwards MC, Liegeois N, Horecka J, DePinho RA, Sprague GF, Jr et al. (1997) Human CPR (cell cycle progression restoration) genes impart a Far- phenotype on yeast cells. Genetics 147: 1063-1076.
4. Figurski DH, Helinski DR (1979) Replication of an origin-containing derivative of plasmid RK2 dependent on a plasmid function provided in *trans*. Proc Natl Acad Sci USA 76: 1648-1652.
5. Hellens R, Mullineaux P, Klee H (2000) A guide to *Agrobacterium* binary Ti vectors. Trends Plant Sci 5: 446-451.
6. Nakagawa T, Suzuki T, Murata S, Nakamura S, Hino T et al. (2007) Improved Gateway binary vectors: high-performance vectors for creation of fusion constructs in transgenic analysis of plants. Biosci Biotechnol Biochem 71: 2095-2100.
7. Quandt J, Hynes MF (1993) Versatile suicide vectors which allow direct selection for gene replacement in gram-negative bacteria. Gene 127: 15-21.
8. Trinick MJ (1980) Relationships amongst the fast-growing rhizobia of *Lablab purpureus, Leucaena leucocephala*, *Mimosa* spp., *Acacia farnesiana* and *Sesbania grandiflora* and their affinities with other rhizobial groups. J Appl Bacteriol 49: 39-53.
9. Töpfer R, Matzeit V, Gronenborn B, Schell J, Steinbiss HH (1987) A set of plant expression vectors for transcriptional and translational fusions. Nucleic Acids Res 15: 5890.
10. Viprey V, Del Greco A, Golinowski W, Broughton WJ, Perret X (1998) Symbiotic implications of type III protein secretion machinery in *Rhizobium*. Mol Microbiol 28: 1381-1389.
11. Wallis JW, Chrebet G, Brodsky G, Rolfe M, Rothstein R (1989) A hyper-recombination mutation in *S. cerevisiae* identifies a novel eukaryotic topoisomerase. Cell 58: 409-419.

Table S2. Primers used in this study

No	Sequence (5' to 3')	Restriction site	Description
1	acaatacatatgaatgtacaac	*Nde*I	Amplification of the 1.64-kb coding region of *nopM* from pSK-*nopM*2500 and insertion into pET28b, generating pET28b-*nopM*
2	gacaagcttgccgctcacagctca	*Hin*dIII	
3	ggagctagcgatagcgctgaggatcgcattact	*Aor*51HI	Site-directed mutagenesis with pSK-*nopM*2500 as template; C338A point mutation in *nopM*, generating pSK-*nopM*(C338A)
4	agtaatgcgatcctcagcgctatcgctagctcc		
5	gaacacgcaacgctaccacag		Amplification of a 2.5-kb fragment containing the *nopM* coding region and the upstream 0.8-kb promoter sequence from genomic DNA of *Rhizobium* sp. NGR234; amplicon digested with *Pst*I and *Xba*I and cloned into pBluescript II SK (+), generating pSK-*nopM*2500
6	ccctctagacaggaacagcaattgacagcc	*Xba*I	
7	cgatgaatgtacaacggatcccggacttgccg	*Bam*HI	Site-directed mutagenesis with pSK-*nopM*2500 as template; generating a *Bam*HI restriction site, resulting in plasmid pSK-*nopM*2500(*Bam*HI)
8	cggcaagtccgggatccgttgtacattcatcg		
9	tttggatccatgaatgtacaac	*Bam*HI	Amplification of the 1.64-kb *nopM* coding region from pSK-*nopM*2500, generating pCAMBIA-*nopM*
10	tttctagatcacagctcaagaccgcgac	*Xba*I	
11	tgctctagaggaacacgcaacgctaccaag	*Xba*I	Amplification of a 2.34-kb *nopM* fragment (with *Xba*I overhangs); identification of mutant strain NGR*nopM*(C338A): amplicon digested by *Aor*51HI into two smaller fragments
12	tgctctagagcctggcataacctgatctcgg		

13	tgaaagcttgcatgtttttccttgagccg	*Hin*dIII	Amplification of the *nopM* coding region and promoter sequence from genomic DNA of *Rhizobium* sp. NGR234; insertion of the 2.08-kb amplicon into pFAJ1702, generating pFAJ-*nopM*
14	acgggtacctcacagctcaagaccgcgac	*Kpn*I	
15	aaggtccaccgcaccatgtccttagag		qRT-PCR; amplification of a *NbCyp71D20* fragment from *N. benthamiana* cDNA
16	aagaattccttgccccttgagtacttgc		
17	aaggtcccgtcttcgtcggatcttcg		qRT-PCR; amplification of a *NbAcre31* fragment from *N. benthamiana* cDNA
18	aagaattcggccatcgtgatcttggtc		
19	aaggtccagtatgcctgggtgcttgac		qRT-PCR; amplification of a *NbEF1α* fragment from *N. benthamiana* cDNA
20	aagaattcacagggacagttccaatacca		
21	tttactagtaatgaatgtacaacggcccggacttg	*Spe*I	Amplification of the *nopM* coding region from pSK-*nopM*2500 and insertion into pESC-leu, generating pESC-*nopM*
22	tttgagctctcacagctcaagaccgcgaccccgaag	*Sac*I	

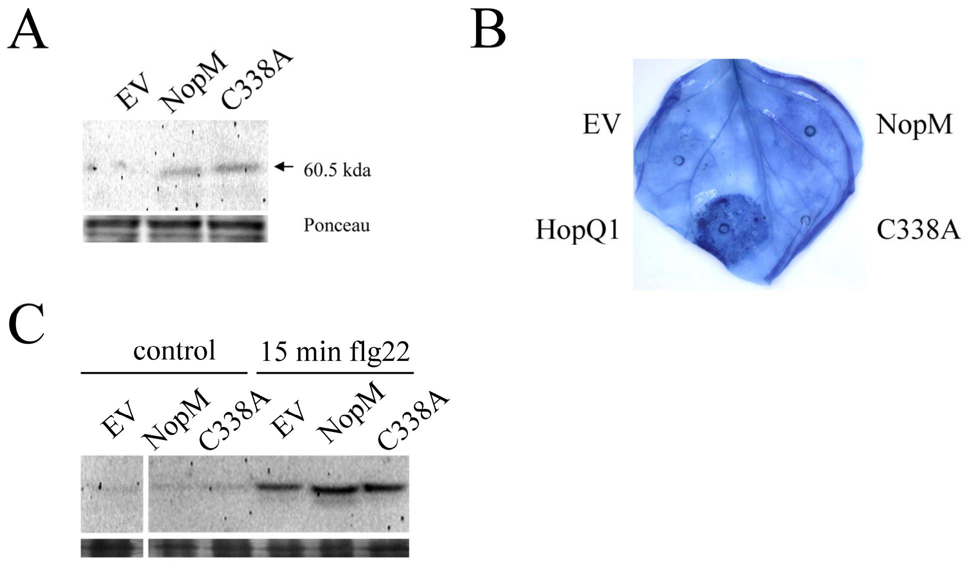

Figure S1 Analysis of *N. benthamiana* expressing NopM and NopM-C338A by immunoblot analysis, trypan blue based cell death staining and MAP kinase activation in response to flg22. (A) Immunoblot analysis of NopM (lane NopM) and NopM-C338A (lane C338A) isolated from infiltrated *N. benthamiana* leaves using the anti-NopM antibodies. Proteins from leaves transformed with *A. tumefaciens* carrying the empty vector pCAMBIA-T were used as a control (lane EV). Ponceau staining illustrates equal loading of protein samples. **(B)** Trypan blue based cell death staining of a representative *N. benthamiana* leaf transiently expressing NopM and NopM-C338A two days post *Agrobacterium* infiltration. Infiltrated zones are marked by circles (EV, infiltration zone with *A. tumefaciens* carrying the empty vector pCAMBIA-T). For comparison, *A. tumefaciens* carrying plasmid pGWB417-HopQ1-myc was also used for infiltration (expression of HopQ1 of *P. syringae* pv. *tomato* DC3000). The experiment was repeated three times with three technical repeats each, yielding similar results. **(C)** MAP kinase activation in response to flg22 peptide treatment in *N. benthamiana*. Leaves expressing NopM (lanes NopM), NopM-C338A (lanes C338A) or controls transformed with the empty vector pCAMBIA-T (lanes EV) were either infiltrated with 1 μM flg22 peptide or mock-treated with BSA/NaCl (control) for 15 min. Extracted proteins were subjected to immunoblot analysis using an anti-p42/44-phospho-ERK antibody, which cross-reacts with activated SIPK (strong upper band) and WIPK (faint lower band). Ponceau staining was used as loading control of protein samples. The experiment was repeated three times with two technical repeats each, yielding similar results.
